# Does a Non-English-Speaking Background Affect the Quality of Bowel Preparation for Colonoscopy?

**DOI:** 10.7759/cureus.116156

**Published:** 2026-09-12

**Authors:** Mathew J Muir, Tien Yew Chern, Natalie Nguyen, Keith Wai Keong Choong, Jessica Rahme, Sharon Lee, Haider Abdulrasool, Ankur Sidhu, Basil D'Souza

**Affiliations:** 1 Department of Surgery, Austin Health, Melbourne, AUS; 2 Department of Surgery, The Northern Hospital, Melbourne, AUS; 3 Department of General Surgery, Lyell McEwin Hospital, Adelaide, AUS

**Keywords:** colonoscopy bowel preparation, colonoscopy preparation, colonoscopy, git endoscopy, language and communication

## Abstract

Background

Bowel preparation quality is critical for colonoscopy diagnostic yield. Whether a non-English-speaking background (NESB) adversely affects preparation quality in Australia is unknown. We aimed to determine whether NESB status is associated with inadequate bowel preparation compared to English-speaking background (ESB) patients, and to identify other clinical predictors.

Methods

A retrospective audit of consecutive patients undergoing elective colonoscopy at the Northern Hospital, Epping, Victoria (September-November 2017). NESB was defined as a primary language other than English. Preparation quality was assessed using the Aronchick scale, dichotomised as adequate (excellent/good/fair) or inadequate. Multivariable logistic regression was performed to identify independent predictors of inadequate preparation.

Results

Of 1,385 patients, 1,129 (81.5%) were ESB and 256 (18.5%) NESB. Professional interpreters were used by 73.0% of NESB patients. Inadequate preparation occurred in 8.9% of ESB and 6.2% of NESB patients; no significant difference was found on univariable (p=0.16) or age-adjusted multivariable analysis (adjusted odds ratio (OR) 0.73, 95% confidence interval (CI) 0.42-1.27; p=0.26). No factor reached statistical significance at the 5% level, although extended bowel preparation (adjusted OR 1.83, p=0.06), morning-list colonoscopy (adjusted OR 1.45, p=0.06), Moviprep (adjusted OR 1.47, p=0.09) and age (adjusted OR 0.88 per 10-year increment, in the direction of lower rather than higher risk with increasing age; p=0.06) approached it; male sex was not associated with inadequate preparation (adjusted OR 1.27, p=0.23).

Conclusions

We found no evidence of an association between NESB status and inadequate bowel preparation at a culturally diverse Australian institution, although the confidence interval remains compatible with both a modest protective and a modest harmful association. High utilisation of professional interpreter services may mitigate language barriers in preparation adherence. In contrast to previous reports, male sex, extended preparation and morning-list timing were not statistically significant independent predictors of inadequate preparation in this cohort, although extended preparation and morning-list timing showed trends consistent with the published literature that this study was underpowered to resolve.

## Introduction

The detection of adenomatous polyps and colorectal cancer is dependent on the quality of the preparation for colonoscopy [1,2]. Inadequate preparation limits the visualisation of the mucosa and any potential lesions, and factors associated with inadequate bowel preparation are well described [3,4]. However, there is little evidence to suggest whether linguistic background influences the quality of bowel preparation in elective colonoscopy. Such patients may experience barriers in communication that affect understanding of bowel preparation instructions, which could impact quality [5,6].

The Northern Hospital in Victoria, Australia, serves a large catchment area that captures a culturally and linguistically diverse community. The hospital is equipped with readily accessible professional interpreters both on-site and through phone service, reducing the language barrier to explain and give instructions on the procedure and bowel preparation.

The primary aim of this study was to determine whether patients from non-English-speaking backgrounds (NESB) were associated with poor bowel preparation compared to patients from English-speaking backgrounds (ESB). The secondary aim was to determine other factors contributing to poor bowel preparation.

## Materials and methods

Study participants

This study was a single-centre, retrospective observational audit conducted at The Northern Hospital, Epping, Victoria, Australia. All consecutive patients undergoing elective colonoscopy over a three-month period from September to November 2017 were screened. Of these 1,418 records, 31 were excluded because no colonoscopy was ultimately performed (n=28) or the record was a duplicate entry (n=3), and a further two were excluded as the patient was aged under 18 years, yielding 1,385 patients for analysis. Language background, age, sex, list timing and Aronchick grade were complete for all analysed patients; no analysed patient lacked an Aronchick grade. Patients who listed English as their primary language were identified and classified as ESB, while patients who spoke a primary language other than English were classified as NESB.

Data collection and statistical analysis

A formal Aronchick grade was not routinely recorded by the original endoscopists; although scoring systems such as the Boston Bowel Preparation Scale were sometimes used, their application was inconsistent and they could not be applied uniformly across the cohort. Bowel preparation was therefore graded retrospectively for every case by a single investigator, who applied the validated Aronchick scale [7] to the endoscopist’s narrative description in the colonoscopy report. Reports describing residual solid or semi-solid stool precluding adequate mucosal visualisation or explicitly recommending an earlier repeat examination on the grounds of preparation quality were classified as inadequate; all other descriptions were assigned excellent, good or fair according to the standard Aronchick definitions. The investigator was not blinded to patient language background, and formal inter-rater reliability was not assessed.

The standard preparation regimens were Prep Kit C (sodium picosulfate/magnesium citrate) (Fresenius Kabi Australia Pty Ltd, Pymble, NSW, Australia) or Moviprep (polyethylene glycol-electrolyte solution) (Norgine Pty Ltd, Frenchs Forest, NSW, Australia), each prescribed over the two days before the procedure and comprising dietary modification (a low-residue diet followed by clear fluids), with the oral laxative taken on the evening before the colonoscopy and a further dose on the morning of the procedure for afternoon lists. Extended preparation was defined as any regimen prescribed over a longer duration than the standard two-day prescription. In practice, this most often comprised extension of dietary modification (a low-residue diet followed by clear fluids) for a further 24 to 48 hours before the standard purgative, with or without an additional purgative dose on the day prior to the procedure. Because extended regimens were prescribed at the discretion of the referring or booking clinician and recorded as free text rather than to a fixed protocol, the precise number of additional purgative doses was not uniformly documented and could not be quantified retrospectively.

Data were manually collected by three clinicians. Patients were dichotomised based on an adequate preparation (grades excellent, good or fair) or inadequate. Descriptive statistics were generated and multivariable logistic regression analysis was conducted using Stata (16.1 StataCorp LLC, College Station, TX). The Pearson χ² test was used for categorical variables and the Mann-Whitney U test for continuous variables, as age was non-normally distributed (Shapiro-Wilk p<0.001).

Multivariable logistic regression was utilised to model the variables associated with inadequate bowel preparation outcomes. Candidate covariates comprised NESB status, age, sex, colonoscopy list timing (morning versus afternoon) and bowel preparation type. Sex, list timing and preparation type were selected a priori on the basis of clinical relevance and previously reported associations. Age was added to the model following peer review, on the grounds that it differed substantially between language groups and is mechanistically linked to colonic transit time, comorbidity burden and mobility; it was modelled as a continuous variable and is reported per 10-year increment. With 117 outcome events and seven model terms, the events-per-variable ratio was 16.7.

Preparation type was modelled as a four-level variable: Prep Kit C / Picoprep (reference), Moviprep, extended preparation, and other or not documented. Patients whose preparation agent was recorded as other or was absent from the record (n=62; 4.5% of ESB and 4.3% of NESB patients) were retained as a distinct category rather than excluded, preserving the full analysed cohort of 1,385. This category comprised 56 patients for whom no preparation agent was documented and six who received a non-standard regimen (a sodium phosphate preparation in three and a bisacodyl-based tablet preparation in three). Two sensitivity analyses were performed to confirm that the primary estimate was not an artefact of this decision.

All model covariates and the outcome were complete for every analysed patient. Some variables were incompletely recorded and were not entered into any model; the most important of these was the presence of a previous colonoscopy, which was undocumented in 225 patients (16.2%). Such variables were used only in descriptive comparisons, restricted to patients with recorded data and reported with their denominators. Adjusted odds ratios are reported with 95% confidence intervals and Wald test p-values. A separate multivariable logistic regression was run in which NESB status was stratified by interpreter use. As a sensitivity analysis, the multivariable model was repeated using an objective endpoint, the need for repeat colonoscopy, in place of the Aronchick grade, to assess whether the primary findings held against a clinically actioned measure of inadequate preparation.

The level of significance was a p-value of less than 0.05. An a priori sample size calculation was performed: to detect an odds ratio of 1.5 for NESB status using a two-tailed test with a significance level of 0.05 and 80% power, a minimum of 584 patients was required, a threshold exceeded by the available cohort of 1,385. This project was reviewed by the Northern Health Human Research Ethics Committee and Research Governance Unit, which determined that it constituted a Quality Improvement and Innovation activity and did not require submission for formal ethical approval (Reference No. ALR 54.2018). All procedures were performed in accordance with the ethical standards of the Declaration of Helsinki (as revised in 2013).

## Results

Patient demographics and characteristics are summarised in Table 1. Of the 1,385 patients who underwent elective colonoscopy during the study period, 1,129 (81.5%) patients were ESB and 256 (18.5%) were NESB. A total of 633 (45.7%) patients were men, and 752 (54.3%) patients were women. NESB patients were older on average than ESB patients (p<0.001), with the median age among NESB patients being 68 years (interquartile range (IQR) 56-75) and among ESB being 59 years (IQR 49-69).

**Table 1:** Baseline characteristics stratified by language background. Comparisons used the Pearson χ² test for categorical variables (χ²: sex 1.93, df 1; list timing 0.36, df 1; preparation type 6.07, df 3) and the Mann–Whitney U test for age (non-normally distributed). The p-value shown against extended bowel preparation tests bowel preparation type as a four-level variable, not extended preparation alone. ESB: English-speaking background; NSEB: non-English-speaking background; IQR: interquartile range.

Characteristic	ESB (n=1,129)	NESB (n=256)	p-value
Age, median (IQR), years	59 (49-69)	68 (56-75)	<0.001
Male sex, n (%)	526 (46.6%)	107 (41.8%)	0.16
Female sex, n (%)	603 (53.4%)	149 (58.2%)	
Morning colonoscopy, n (%)	572 (50.7%)	135 (52.7%)	0.55
Afternoon colonoscopy, n (%)	557 (49.3%)	121 (47.3%)	
Extended bowel preparation, n (%)	97 (8.6%)	19 (7.4%)	0.11
Moviprep, n (%)	310 (27.5%)	90 (35.2%)	
Prep Kit C / Picoprep, n (%)	671 (59.4%)	136 (53.1%)	
Other or not documented preparation, n (%)	51 (4.5%)	11 (4.3%)	
Interpreter use, n (%)	NA	187 (73.0%)	NA

Twenty-four different primary languages were identified among the NESB cohort. An interpreter was used by 187 NESB patients (73.0%) for their clinic consultation and consent process. In total, 707 (51.0%) patients had a colonoscopy on a morning list, while 678 (49.0%) had their colonoscopy in the afternoon. An extended bowel preparation was used in 116 patients (8.4%). Aronchick scores stratified by language background are summarised in Table 2.

**Table 2:** Aronchick scores among ESB and NESB patients. ESB: English-speaking background; NSEB: non-English-speaking background.

Aronchick Score	ESB (n=1,129)	NESB (n=256)
Excellent, n (%)	392 (34.7%)	98 (38.3%)
Good, n (%)	372 (32.9%)	79 (30.9%)
Fair, n (%)	264 (23.4%)	63 (24.6%)
Inadequate, n (%)	101 (8.9%)	16 (6.2%)

Inadequate bowel preparation occurred in 101 (8.9%) ESB patients and 16 (6.2%) NESB patients, with no statistically significant difference on univariable (p=0.16) or age-adjusted multivariable analysis (adjusted OR 0.73, 95% CI 0.42-1.27; p=0.26). On multivariable analysis, no covariate reached statistical significance at the 5% level, although extended bowel preparation compared with Prep Kit C / Picoprep (adjusted OR 1.83, 95% CI 0.98-3.42; p=0.06), morning-list compared with afternoon-list colonoscopy (adjusted OR 1.45, 95% CI 0.98-2.14; p=0.06) and Moviprep compared with Prep Kit C / Picoprep (adjusted OR 1.47, 95% CI 0.94-2.28; p=0.09) approached it; male sex was not associated with inadequate preparation (adjusted OR 1.27, 95% CI 0.86-1.86; p=0.23).

Age was not independently associated with inadequate preparation (adjusted OR 0.88 per 10-year increment, 95% CI 0.78-1.01; p=0.06), and its addition did not materially alter the estimate for NESB status (likelihood ratio test χ²=3.42, df 1, p=0.06).

Patients receiving other or undocumented preparation were not at increased risk of inadequate preparation (adjusted OR 1.13, 95% CI 0.44-2.94; p=0.80), and the estimate for NESB status was stable across sensitivity analyses varying the handling of this group: excluding these patients, adjusted OR 0.70 (95% CI 0.40-1.25; n=1,323); collapsing them into the reference category, adjusted OR 0.73 (95% CI 0.42-1.27; n=1,385). The results of the multivariable logistic regression analysis are presented in Table 3.

**Table 3:** Multivariable logistic regression analysis of factors associated with inadequate bowel preparation. Adjusted odds ratios, 95% confidence intervals and p-values are derived from multivariable logistic regression (Wald test). Bowel preparation type was modelled as a four-level categorical variable with Prep Kit C / Picoprep as the reference. Wald z-statistics: NESB −1.12, age −1.87, male sex 1.21, morning-list 1.85, extended preparation 1.91, Moviprep 1.69, other or not documented preparation 0.25. ESB: English-speaking background; NSEB: non-English-speaking background.

Variable	Inadequate prep n (%)	Adjusted OR	95% CI	p-value
NESB (vs ESB)	16 (6.2%)	0.73	0.42–1.27	0.26
Age (per 10-year increment)	—	0.88	0.78–1.01	0.06
Male sex (vs female)	60 (9.5%)	1.27	0.86–1.86	0.23
Morning colonoscopy (vs afternoon)	68 (9.6%)	1.45	0.98–2.14	0.06
Extended bowel preparation (vs Prep Kit C / Picoprep)	14 (12.1%)	1.83	0.98–3.42	0.06
Moviprep (vs Prep Kit C / Picoprep)	38 (9.5%)	1.47	0.94–2.28	0.09
Other or not documented preparation (vs Prep Kit C / Picoprep)	5 (8.1%)	1.13	0.44–2.94	0.80

There was no significant difference in the likelihood of inadequate bowel preparation based on whether an interpreter was utilised or not within the NESB cohort when compared with the ESB cohort (ESB vs NESB with interpreter: p=0.49; ESB vs NESB without interpreter: p=0.27). Consistent with the primary model, male sex, extended preparation and morning-list timing were not independently associated with inadequate preparation in this interpreter-stratified model (Table 4).

**Table 4:** Multivariable logistic regression analysis of factors associated with inadequate bowel preparation, stratified by interpreter use. Adjusted odds ratios, 95% confidence intervals and p-values are derived from multivariable logistic regression (Wald test). Wald z-statistics: NESB with interpreter −0.69, NESB without interpreter -1.11, male sex 1.21, morning-list 1.85, extended preparation 1.89, Moviprep 1.68, other or not documented preparation 0.25, age -1.87. ESB: English-speaking background; NSEB: non-English-speaking background.

Group or variable	Inadequate prep n (%)	Adjusted OR	95% CI	p-value
ESB	101 (8.9%)	1.00	Reference	
NESB with interpreter	13 (7.0%)	0.81	0.44-1.49	0.49
NESB without interpreter	3 (4.3%)	0.51	0.16-1.67	0.27
Male sex (vs female)	60 (9.5%)	1.27	0.86-1.86	0.23
Morning colonoscopy (vs afternoon)	68 (9.6%)	1.45	0.98-2.14	0.06
Extended bowel preparation (vs Prep Kit C / Picoprep)	14 (12.1%)	1.82	0.98-3.40	0.06
Moviprep (vs Prep Kit C / Picoprep)	38 (9.5%)	1.46	0.94-2.28	0.09
Other or not documented preparation (vs Prep Kit C / Picoprep)	5 (8.1%)	1.13	0.43-2.93	0.81
Age (per 10-year increment)	—	0.88	0.78-1.01	0.06

A repeat colonoscopy was required in 69 patients (5.0%). In the sensitivity model, no covariate was independently associated with the need for a repeat colonoscopy: NESB status (adjusted OR 1.11, 95% CI 0.59-2.09; p=0.75), male sex (adjusted OR 1.19, 95% CI 0.73-1.94; p=0.48), morning-list colonoscopy (adjusted OR 1.23, 95% CI 0.75-2.01; p=0.42), extended bowel preparation (adjusted OR 1.55, 95% CI 0.73-3.28; p=0.26) and age (adjusted OR 0.94 per 10-year increment, 95% CI 0.79-1.12; p=0.48).

These findings are consistent with the primary Aronchick analysis (Table 5). As the reasons for repeat colonoscopy were not consistently recorded, this endpoint reflects all-cause repeat colonoscopy; 12 patients had adequate preparation at the index procedure and were likely repeated for reasons unrelated to preparation quality.

**Table 5:** Sensitivity analysis: multivariable logistic regression of factors associated with the requirement for a repeat colonoscopy. Adjusted odds ratios (ORs), 95% confidence intervals (CI) and p-values are derived from multivariable logistic regression (Wald test). Wald z-statistics: NESB 0.32, male sex 0.71, morning-list 0.81, extended preparation 1.14, Moviprep -1.23, other or not documented preparation -0.13, age -0.70.

Variable	Repeat n (%)	Adjusted OR	95% CI	p-value
NESB (vs ESB)	13 (5.1%)	1.11	0.59–2.09	0.75
Male sex (vs female)	34 (5.4%)	1.19	0.73–1.94	0.48
Morning colonoscopy (vs afternoon)	38 (5.4%)	1.23	0.75–2.01	0.42
Extended bowel preparation (vs Prep Kit C / Picoprep)	9 (7.8%)	1.55	0.73–3.28	0.26
Moviprep (vs Prep Kit C / Picoprep)	14 (3.5%)	0.67	0.35–1.27	0.22
Other or not documented preparation (vs Prep Kit C / Picoprep)	3 (4.8%)	0.92	0.28–3.07	0.90
Age (per 10-year increment)	-	0.94	0.79–1.12	0.48

## Discussion

Australia’s ageing population is increasingly ethnically and linguistically diverse [8,9], and it is important to evaluate how this demographic shift may influence how health services can improve delivery of treatment and respond to different healthcare beliefs, practices and cultural needs to ensure equitable access.

The most common languages after English were Mandarin, Arabic, Vietnamese and Cantonese [10]. According to the 2021 Australian census, 37.6% of households in Northeast Melbourne used a non-English language at home, most commonly Italian, Arabic, Greek, Mandarin and Macedonian [11]. Previous Australian studies have reported poorer hospital outcomes for NESB patients, including longer psychiatric inpatient length of stay [12] and higher rates of admission for acute myocardial infarction [13]. Furthermore, Basic et al. found that migrants from non-English-speaking countries admitted to an acute geriatric service were no more likely than English-speaking patients to die in hospital overall, although the subgroup unable to speak English did have higher in-hospital mortality (11.5% versus 7.2%, p=0.02) [14].

In this study, no statistically significant difference in the bowel preparation quality was observed between ESB and NESB patients undergoing elective colonoscopy. This may reflect the high utilisation of professional interpreters in this study cohort, with 73.0% receiving their support through the consenting process. The Northern Hospital supports this with over 40 in-house interpreters, catering for the most common languages spoken in the catchment, and there is 24-hour availability for less commonly spoken languages through external agencies. There is also the possible impact of a number of NESB patients who are competent in English despite it not being their primary language, or who have family members that can support them through interpreting bowel preparation instructions for them. Multilingual instruction sheets and referrals to the interpreter service are also routinely provided. The low rate of inadequate preparation among NESB patients who did not use an interpreter should not, however, be read as evidence that proceeding without an interpreter is safe. These 69 patients are a selected rather than a random sample, most plausibly comprising those with sufficient functional English or reliable family support, and the estimate rests on only three events (4.3%; p=0.27 versus ESB patients). Their median age was comparable to that of NESB patients who did use an interpreter (69 versus 68 years), so the difference is unlikely to be explained by a younger or fitter case mix.

In contrast to several previous studies, male sex, extended bowel preparation and morning-list timing were not independently associated with poorer preparation quality in our cohort, although each showed a non-significant trend in the expected direction. Male sex has nonetheless been reported as an independent predictor of poor bowel preparation quality in some Western populations; Lebwohl et al. [3] found that men were 1.4 times more likely to have poor bowel preparation in a study of 10,921 colonoscopies. In an Asian cohort of 501 patients, Chan et al. [15] found male sex was not an independent predictor, with lower education level, appointment waiting time beyond 16 weeks and non-adherence to preparation instructions being the significant factors. Proposed explanations for this reported association include differences in physical health, health perceptions, illness reporting, engagement in preventive healthcare and sociocultural factors [16-19]. The absence of a significant association in our data may reflect the lower event rate under the Aronchick definition and limited statistical power for these secondary endpoints.

The trend towards poorer bowel preparation among patients on the morning list, although not statistically significant in this cohort, is biologically plausible and likely relates to the difference in timing and regimen between morning and afternoon patients. At our institution, morning cases complete their bowel preparation the night before, whereas afternoon cases receive a further dose on the morning of the colonoscopy, likely contributing to the superior quality of bowel preparation in the afternoon group.

In a study of 317 patients that utilised polyethylene glycol bowel preparation, Church [20] demonstrated that same-day administration had significantly better quality outcomes compared with day-before regimens using the same quantity of preparation. This phenomenon was also observed by Parra-Blanco et al. [21] in a randomised controlled trial of 177 patients, which demonstrated improved bowel preparation with same-day regimens, as well as a higher rate of identifying flat colonic lesions.

Although extended bowel preparation showed only a non-significant trend towards higher rates of poor preparation, this apparent paradox may arise because these patients had poor preparation in the past and therefore required additional bowel preparation this time. Consistent with this, patients receiving extended preparation were significantly more likely to have a documented previous colonoscopy than those receiving any other regimen (57 of 95 with recorded data, 60.0%, versus 485 of 1,065, 45.5%; p=0.009). The dataset did not, however, record the quality of preparation at that previous procedure, so the proportion of extended-preparation patients with previously documented inadequate preparation cannot be derived; confounding by indication therefore remains a supported but unproven explanation. Increased colonic transit time may also contribute to poorer preparation in these patients. Factors that contribute to increased colonic transit times include patients’ mobility status and comorbidities, in particular Parkinson’s disease and diabetes [4]. Age, an imperfect proxy for these factors, was not independently associated with inadequate preparation in this cohort and, if anything, trended in the opposite direction (adjusted OR 0.88 per decade). This may in part reflect confounding by clinician behaviour, in that older patients and those with recognised risk factors were more likely to be prescribed extended preparation or given additional counselling, such that age in this dataset may partly index preparation intensity rather than physiology alone. This contrasts with Lebwohl et al., who reported increasing age to be associated with suboptimal preparation (odds ratio 1.09 per 10 years) in a larger North American cohort [3].

Limitations

This was a single-centre study and although the findings from this study are informative, the demographics of Melbourne’s northern suburbs may limit the generalisability of these results to other communities. Our institution is also atypical in its interpreter capacity, maintaining more than 40 in-house interpreters with 24-hour external agency coverage and achieving 73.0% uptake among NESB patients. A null result obtained under these conditions should not be extrapolated to services with limited, delayed or predominantly telephone-based interpreter access, where a language barrier may remain an active determinant of preparation quality.

More fundamentally, this was a retrospective observational study and cannot establish causation. Our null result should not be read as evidence that language barriers do not affect bowel preparation quality; given the level of interpreter uptake described above, it is at least as consistent with a language barrier that has been effectively mitigated as with one that was never present, and this design cannot distinguish between those two explanations.

Several potentially important factors such as level of education, literacy levels and socioeconomic status were not included in the analysis, which may have affected the quality of bowel preparation outcome. NESB as a cohort definition is likely to be an oversimplification, as English fluency among the NESB patients was not routinely recorded.

Another limitation relates to the documentation of bowel preparation quality. As a single standardised scoring system to document bowel preparation was not in use at our institution during the study period, the quality of preparation had to be reassessed retrospectively for each case using the validated and standardised Aronchick scale based on the endoscopist’s documentation in the colonoscopy report, which introduces the possibility of misclassification and observer subjectivity. Because a single investigator performed this re-grading without blinding to patients’ language background, and formal inter-rater reliability was not assessed, both misclassification and detection bias are possible. Future prospective studies should use assessors independent of, and masked to, patient demographics, with reported inter-rater agreement.

Inadequate preparation occurred in only 117 patients (8.4%). While this event count was sufficient for the primary comparison, for which the study was powered, it constrains precision throughout the secondary and subgroup analyses: the confidence intervals around the estimates for sex, list timing and preparation type are correspondingly wide, and these results should not be read as excluding modest associations for those covariates. The interpreter-stratified analysis is more limited still, resting on 13 events among NESB patients who used an interpreter and three among those who did not. The conservative definition of inadequacy used in this study may also underestimate lesser degrees of suboptimal but clinically acceptable bowel preparation.

Furthermore, other potential confounders including patient comorbidities, mobility status and medications that may affect colonic transit times were not extracted for this cohort and could not be entered into the multivariable model. Residual confounding by these omitted variables therefore cannot be excluded, and prospective capture of comorbidity data is a priority for future work. Although age was included in the final model, it is an imprecise surrogate for these factors and its inclusion does not remedy their absence. The bowel preparation agent was also not recorded in 4.5% of cases, a category that necessarily conflates genuinely non-standard regimens with simple documentation failure; these patients were retained in the model as a separate category, and sensitivity analyses confirmed that their handling did not alter the primary finding.

## Conclusions

In this single-centre Australian cohort, we found no evidence of an association between non-English-speaking background and poorer colonoscopy bowel preparation quality, and no covariate was statistically significantly predictive of inadequate preparation. The confidence interval around the adjusted estimate (0.42 to 1.27) remains compatible with both a modest protective and a modest harmful association, and this result should not be interpreted as demonstrating the absence of an effect. The high utilisation of professional interpreter services may help mitigate language-related barriers to preparation adherence in a linguistically diverse population. Given Australia’s increasing cultural and linguistic diversity, further research, including qualitative, patient-reported evaluations, is warranted to confirm these findings and to ensure equitable colonoscopy outcomes across all backgrounds.
